# Cervical Spine Manipulations: Role of Diagnostic Procedures, Effectiveness, and Safety from a Rehabilitation and Forensic Medicine Perspective: A Systematic Review

**DOI:** 10.3390/diagnostics12051056

**Published:** 2022-04-23

**Authors:** Andrea Bernetti, Raffaele La Russa, Alessandro de Sire, Francesco Agostini, Stefania De Simone, Giacomo Farì, Giorgia Viola Lacasella, Gabriele Santilli, Stefania De Trane, Michele Karaboue, Pierangela Ruiu, Massimiliano Mangone, Massimiliano Leigheb, Valter Santilli, Pietro Fiore

**Affiliations:** 1 Department of Anatomy, Histology, Forensic Medicine and Orthopedics, Sapienza University, 00185 Rome, Italy; andrea.bernetti@uniroma1.it (A.B.); francesco.agostini@uniroma1.it (F.A.); pierangela.ruiu@uniroma1.it (P.R.); massimiliano.mangone@uniroma1.it (M.M.); valter.santilli@uniroma1.it (V.S.); 2 Italian Society of Physical and Rehabilitation Medicine (SIMFER), 00198 Rome, Italy; pietro.fiore@unifg.it; 3 Department of Clinical and Experimental Medicine, University of Foggia, 71122 Foggia, Italy; raffaele.larussa@unifg.it (R.L.R.); stefania.desimone@unifg.it (S.D.S.); michele.karaboue@unifg.it (M.K.); 4 Department of Medical and Surgical Sciences, University of Catanzaro “Magna Graecia”, 88100 Catanzaro, Italy; 5 Department of Basic Medicine, Neuroscience, and Sense Organs, University of Bari “Aldo Moro”, 70124 Bari, Italy; dr.giacomofari@gmail.com; 6 Department of Medicine and Health Sciences, University of Molise, 86100 Campobasso, Italy; g.lacasella@studenti.unimol.it; 7 Department of Surgical and Medical Sciences and Translational Medicine, Sapienza University, 00185 Rome, Italy; gabriel.santilli@uniroma1.it; 8 Neurological Rehabilitation Unit, Clinical Scientific Institutes Maugeri IRCCS, 70124 Bari, Italy; stefania.detrane@icsmaugeri.it; 9 Orthopaedics and Traumatology Unit, “Maggiore della Carità” Hospital, Department of Health Sciences, University of Eastern Piedmont, 28100 Novara, Italy; massimiliano.leigheb@med.uniupo.it

**Keywords:** cervical spine manipulations, manipulative therapy, cervical artery dissection, spine, side effects, rehabilitation, forensic medicine

## Abstract

Background: Cervical spine manipulations (CSM) have been performed for centuries and are a widely practiced intervention to manage cervical spine musculoskeletal disorders. We aimed to perform an overview of the literature concerning the effects and the adverse events of CSM in the Physical and Rehabilitation Medicine (PRM) field with a forensic medicine perspective. Methods: A search in the scientific literature (PubMed, Google Scholar, PEDro and Cochrane) was carried out from inception until October 2020. Results: Fourteen articles were included in this narrative summary. The possible development of side effects requires a careful mandatory balance of benefits and risks even when there is an indication for this approach. Moreover, a qualified professional is essential to perform CSM–a non-invasive therapeutic procedure that can be potentially harmful. Conclusions: In conclusion, it is essential to perform the diagnosis, to treat, and to manage complications within the PRM field, both for the reduction of malpractice claims and, most importantly, for the safety of the patient.

## 1. Introduction

According to the World Health Organization, spinal manipulative therapy “includes all procedures where the hands or mechanical devices are used to mobilize, adjust, manipulate, apply traction, massage, stimulate, or otherwise influence the spine and paraspinal tissues with the aim of influencing the patient’s health” [1].

Spine manipulations are passive forced mobilizations which tend to bring articular elements beyond their passive range of motion [2]. Cervical spine manipulations (CSMs), a common type of spinal manipulative therapy, have been performed for centuries [3] and remain a widely practiced intervention [4]. These therapeutic procedures generally consist of high-velocity, low-amplitude approaches [5], involving single or combined movements of rotation, latero-flexion, flexion, or extension of a definite vertebral segment [2].

CSMs are implemented to manage cervical spine musculoskeletal disorders, particularly mechanical neck pain, stiffness, cervicogenic headaches, and cervical radiculopathy [3,6]. They are usually applied by different healthcare professionals which, depending on the country, can be medical doctors, physiotherapists, practitioners in osteopathy, and chiropractors [2,7].

However, an adequate clinical assessment of the indication for CSMs is mandatory to avoid interventions for inappropriate clinical conditions [6,8]. Physical and rehabilitative medicine (PRM) professionals have the role to assess the subject, making a differential diagnosis, and always balancing benefits and potential risks of the procedure [9,10,11,12,13].

Despite their great popularity worldwide [9,14], CSMs benefits have not yet been clearly established [7]. This is probably due to the large differences among CSM techniques–which are often poorly described in literature, as also observed by Haldeman et al. [14]–and because of their potential association with serious adverse events [6]. CSMs could in fact determine various types of complications (i.e., neurovascular, neurological, and musculoskeletal [2]. Cerebrovascular insult represents one of the most serious side effects [15]. In particular, cervical artery dissection (CAD), which comprises carotid and vertebral artery dissection (VAD) [8], is one of the most catastrophic adverse events associated with cervical manipulative therapy. Stroke is a rare condition in the young adult population, with less than 5% of all strokes occurring in people younger than 45 years [16]. CAD accounts for up to 25% of all ischemic strokes in people under 55 years and 2% of all ischemic strokes [17]. Stroke is caused either by the propagation of a thrombus from the dissected arterial segment or by severe dissection-induced arterial stenosis and secondary ischemia [18].

Therefore, taking into account the progressive and widespread clinical use of this therapeutic procedure, we aimed to perform a systematic review of the literature concerning the role of diagnostic procedures, effectiveness, and the potential adverse events of CSMs in a rehabilitation and forensic medicine perspective.

## 2. Materials and Methods

A systematic search in the scientific literature through the PubMed, Google Scholar, PEDro, and Cochrane databases was carried out from inception until October 2020, using Mesh and free-text terms as: “Cervical Spine Manipulations”, “Cervical Spine Manipulative Therapy” and “Manipulative Therapy” combined with “Adverse events”, “Side Effects”, “Cervical Artery Dissection”, “Vertebral Artery Dissection”, “Carotid Artery Dissection”, and “Stroke”. The exclusion criteria were as follows: full text not available; articles not in English language.

The authors independently performed the search and removed duplicate records. Then, data extraction was independently performed and the inconsistencies were overcome by the comparison of the data and debate. A summary of the selected articles was performed.

## 3. Results

**Figure 1:**
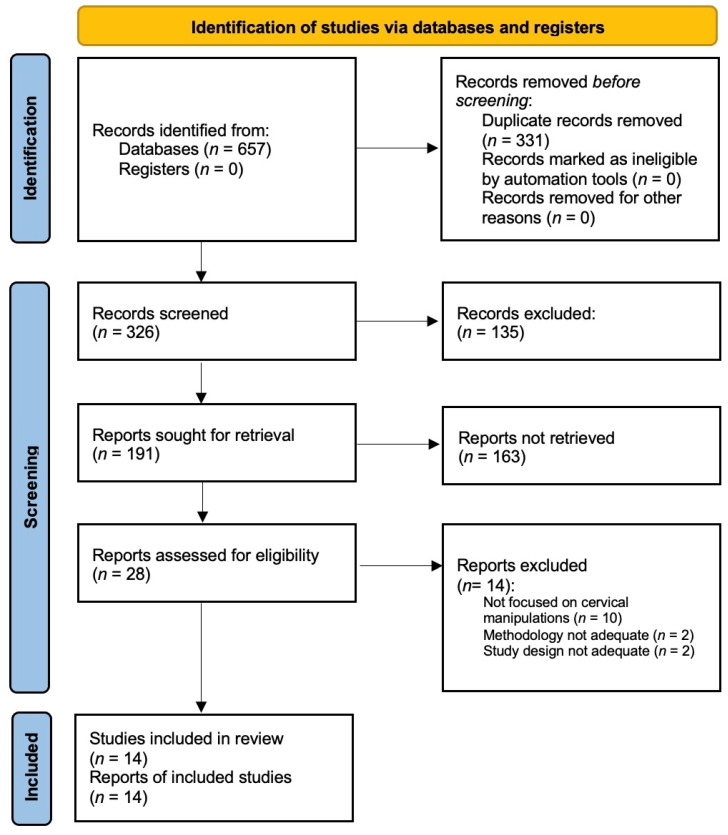
PRISMA 2020 Flow Diagram.

The research resulted in 14 articles, which were divided into two main groups: case report/case series group and clinical studies group (see Figure 1 for further details).

### 3.1. Case Reports and Case Series

Milkkelsen et al. [19] presented a case report of a 37-year-old female who, immediately after chiropractic CSM therapy, developed a bilateral VAD, responsible for the embolic occlusion of her basilar artery. The subject, treated with endovascular therapy, presented–at six months–minor sensory and cognitive deficits. They pointed out that VAD can complicate into a basilar artery thrombosis, and stressed how a prompt diagnosis and the use of advanced endovascular procedures are essential to ensure a long term favorable neurological outcome.

Orsini et al. [20] described the clinical case of a 34-year-old woman who underwent a CSM performed by a chiropractor. The procedure was followed by a traumatic bilateral VAD which resulted in vertebrobasilar stroke, presenting with tetraplegia and severe aphasia. They described the benefits of a late mechanical thrombectomy that the patient underwent 31 h after symptom onset, leading to a partial recovery of the deficits.

Tinel et al. [2] reported the case of a 34-year-old man–an active smoker with a 10-year history of migraine–who underwent a cervical manipulation. The procedure was followed, seven hours later, by an alternate syndrome, with a right side sensory-motor deficit, cerebellar and pyramidal syndrome, and left side cranial nerves impairment. These manifestations resulted from basilar trunk thrombosis and left vertebral artery dissection, treated with thrombolysis, and followed by eight months of neurorehabilitation.

Jeong et al. [21] described the case of a subject who experienced a left posterior inferior cerebellar artery infarction and a left VAD two weeks after a CSM by a chiropractor. The man, treated with antiplatelets therapy, was discharged three weeks later and presented no neurological sequelae. They therefore suggested that patients with cerebellar dysfunctions who have undergone a recent cervical chiropractic manipulation should be assessed for a VA injury, which may minimize the poor prognosis of cerebellar infarction.

Horn [22] published a case report of a 34-year-old man who underwent chiropractic CSM for the treatment of a persistent headache, dizziness, and neck stiffness, which, immediately after the procedure, developed a locked-in syndrome (LIS).

Ke et al. [9] presented the clinical case of a 36-year-old man with a LIS consequent to bilateral VAD, developed after CSM and treated by arterial embolectomy. They sustain that, with regard to the potential severe side effects related to CSM, a regular and rigorous assessment is needed in addition to obtaining informed consent to the procedure. Specific radiological investigations (duplex ultrasonography and magnetic resonance imaging) should be implemented to evaluate the risk of side effects and vascular vulnerability. Moreover, an adequate training of practitioners may also contribute to reducing skill-related adverse events.

Povlsen et al. [23] illustrated the clinical case of a 36-year-old woman who, the day after a chiropractic CSM, developed an incomplete LIS due to the occlusion of the perforating arteries from the basilar artery to the pons.

Hufnagel et al. [10] analysed 10 cases of patients aged between 27 and 46 that developed ischemic stroke secondary to vertebral or carotid artery dissection after chiropractic CSM. They investigated risk factors, neurological impairments, neuroradiological findings, and long-term outcomes. Moderate risk factors were present in five patients. Symptom manifestations were detected from immediately after to two days after CSM. Nine out of ten patients presented severe residual neurological impairments. The long-term outcome ranged from no impairments to very severe impairments with total functional dependency. Given the unpredictability and severity of possible complications, they stressed the importance to carefully evaluate the benefit-risk ratio for each CSM application and to obtain patients’ informed consent for the procedure.

Haldeman et al. [14] analysed 64 medical legal cases of stroke temporally associated with CSM. It emerged that stroke–and particularly vertebrobasilar dissection–should be regarded as an unpredictable complication of any neck movement, including CSM; it can occur at any point of the procedure, with every method of treatment and regardless of the number of manipulations. Moreover, they underlined that a sudden acute and unusual neck/head pain could represent a dissection in progress and could be the reason for seeking manipulative therapy, which served as additional final insult to the vessel and could result in an ischemia.

Albuquerque et al. [18] examined 13 cases of patients presenting dissection of cervical and cranial segments of vertebral and carotid arteries a few hours to a few days after chiropractic manipulations. These injuries were managed with medications or through endovascular stenting and cranial surgery where needed. The follow-up showed that a significant percentage of the patients remained permanently disabled or died (three had irreversible neurological impairments and one died of a massive cerebellar stroke) while nine patients showed a full recovery.

### 3.2. Clinical Studies

Reuter et al. [4] analysed the clinical pattern of 36 VAD cases associated with chiropractic neck manipulation reported over three years in Germany. Out of the 36 patients studied, 55% had VAD 12 h after the manipulation, 90% of which developed focal neurological signs, and among these, the 11% presented a reduced level of consciousness. At discharge, 50% of the total had focal neurological deficit, one died, and one remained in a persistent vegetative state. Risk factors were found in only 25% of them.

Cagnie et al. [24] assessed the frequency of complications following spinal manipulations, including verifying their predictability. They observed relatively common minor side effects that were benign in nature (headache, local and radiating discomfort, stiffness, and fatigue), short-lasting and more likely to affect women (gender is the only predicting variable that showed a statistical significance). They emphasized the importance to differentiate and inform patients susceptible to side effects.

The same authors [15] also examined cerebral blood flow changes after CSM using one-day split-dose Technetium 99 m–ethylcysteinate dimer single photon emission computed tomography. After manipulation, a hypoperfusion of the anterior cerebellar lobe was identified, which could explain the manifestation of some side effects like nausea, headache, or dizziness after CSM.

Main characteristics of the selected articles are reported in Table 1 and Table 2.

## 4. Discussion

The examined articles revealed the high probability of an association between cervical spine manipulative therapy and related complications. Both major and minor adverse events have been described; among these, cerebrovascular insults, and mainly VAD, emerged to be one of the most severe.

It should be noted that the correlation of cervical manipulation with VAD and subsequent ischemic stroke showed to have strong evidence to support the cause-and-effect relationship between cervical manipulation and VAD and subsequent stroke [25]. Furthermore, a case control study performed by Rothwell et al. [26] confirmed that for every 100,000 persons aged <45 years receiving cervical manipulation, approximately 1.3 would develop VAD or occlusion attributable to manipulation within one week. Moreover, they claim that the acceptable level of risk associated with cervical manipulation must be balanced with the evidence of therapeutic efficacy. A great uncertainty on the incidence of CSMs-related complications has been observed by the American Heart Association Stroke Council [27]. Indeed, Biller et al. [27] reviewed the state of evidence on the diagnosis and management of CAD and its association with cervical manipulations, reporting a high heterogeneity in the current evidence.

A variability in the typology of reported adverse events has been detected, with major side effects being more documented than minor ones [28]. However, despite the incidence of major complications being considered low [27,29], numerous studies reporting these events appear to be currently available in literature. Moreover, they are deemed to be under-reported [6].

Minor adverse events, despite the more frequent presentation, are less described in literature. Mainly case reports, small case series [4,14], surveys, or reviews are documented, underpinning the underestimation of CSMs-related side effects [28]. The described variability in incidence reports mainly correlates to a general underestimation of adverse events, especially the minor ones, which given their nature [26] are probably left underdiagnosed; they might be, in most of the cases, neither correlated to CSMs nor documented. It should be also considered that CSMs are often performed both in public and private outpatient settings, making monitoring of related complications even more difficult. Furthermore, their frequency is likely to increase [4] as a consequence of the progressive use of this procedure [14], performed by an ever-growing number of healthcare professional categories [2,7].

As highlighted in several analysed articles [2,4,9,10,14,18,19,20,21,22], CSMs could lead to major irreversible, severely disabling, or even lethal adverse events. Hence, a proper assessment prior to the intervention is essential. The clinician has the role and responsibility to evaluate the patient [9,29] through proper anamnesis, physical examination, and diagnostic techniques, giving the indication–when and if needed–to CSM therapy. However, establishing the indication to the procedure is not sufficient to prevent complications [8].

The clinician should necessarily assess comorbidities, current therapy, identifying red flags, and eventual contraindications, always balancing therapeutic efficacy with potential risks [10]. Differential diagnosis plays an essential role in distinguishing musculoskeletal complaints from ones of other nature, and especially recognizing red flags of a pre-existing VAD [30]. CAD diagnosis can also be supported by ultrasonography, computed tomographic angiography, and magnetic resonance imaging with magnetic resonance angiography, as suggested by Biller et al. [27].

An appropriate medical history taking [17] and screening tools use should drive the pre-manipulative phase [31]; screening tools application is however still debated and remains controversial [8,18,29,31].

As suggested by several authors [9,10,26,27,31], patient information about the association between cervical manipulations and adverse events, together with acquisition of informed consent, should become common practice before performing the procedure.

A skilled professional, as also indicated by Ke et al. [9], is required to perform a non-invasive therapeutic procedure that can be potentially harmful [32], as described by some cases of inadequate application of CSM, such as a documented case of self-manipulation of the neck [30] as well as a described case of a neck massage of a young male (30-year-old) in a massage which resulted in VAD [16].

With this regard, Puentedura et al. underlined that, among healthcare categories, chiropractic professionals appeared to be the most involved in such reported adverse events [8].

It is important to improve the knowledge of health operators about the risks of cervical spine manipulation, especially for non-medical professionals (such as chiropractors) [7]. These maneuvers should be done in a protected place, such as PRM wards, where complications can possibly be properly managed. Moreover, patients must be informed about the risks of cervical manipulation, in particular about the statistical association with cervical manipulations before undergoing cervical spine manipulation [24,25,26,27,28,29,30,31,32,33].

Informed consent is a fundamental act of medical activity: it constitutes the free acceptance of the patient of the medical treatment. The doctor must inform the patient about methods of execution, benefits, possible side effects, reasonably foreseeable risks, and therapeutic alternatives. Law 219/2017 establishes that consent should be written. In the past, informed consent was mandatory only before proceeding with the following procedures: blood transfusion, participating in clinical trials, for the manipulation of sensitive data, for transplants, radiation treatments, and HIV tests. An informed consent form is a full-fledged judicial tool (a generic or incomplete consent form can harm the doctor) [34]. Therefore, informed consent has a dual value, both as an ethical obligation as well as a legal proof to avoid litigation. So, informed consent, acquired in the ways and with the tools most suited to patient conditions, must always be documented in writing or by video recordings or, for disabled people, using special devices. Furthermore, the doctor has to enclose the consent in the medical record.

Indeed, the basis of the doctor-patient relationship is a clear communication, also to prevent medico-legal issues [35]. Malpractice claims linked to neurological complications (such as stroke, CAD, and VAD [36]) are one of the most costly and prevalent [37], contrary to those in the Physical and Rehabilitation Medicine field, which are minor relative to the size of the specialty [38]. There was a significant medico legal burden associated with a serious long-term injury and need for life-long care as in the case of brain damage as compared with death as an outcome [39].

This study has some limitations, represented by a difficulty in performing further type of analysis because of the features of the topic, literature characteristics in relation to this subject, and by the evidence currently present in literature.

However, to the best of our knowledge, this is the first study investigating serious adverse events of CSMs, despite inherent limitations, and might be a cornerstone for the medical forensic implications in the countries where this approach can only be carried out by a physician or under his/her supervision.

This paper could be considered as a best practice similar to others recently published in the field of physical and rehabilitation medicine [40]. In conclusion, it is essential to perform the diagnosis, to treat, and to manage complications within the PRM field, both for the reduction of malpractice claims and, most importantly, for the safety of the patient [41,42,43,44].

## 5. Conclusions

Cervical manipulations are rehabilitative approached with positive effects and, at the same time, potential complications, such as severely disabling, or other adverse events.

Taken together, the findings of the present systematic review showed the potential adverse events of CSM, highlighting that the scientific literature reported only a small part of the number of adverse events (i.e., cerebrovascular insults) occurring in the clinical practice. In this scenario, we might conclude that PRM physicians should perform CSM only after an adequate diagnosis and with a monitoring of the potential adverse events.

## Figures and Tables

**Table 1:** Characteristics and summary of the analysed studies.

Authors, Year	Type of Study	Summary
Milkkelsen et al., 2015	Case report	Clinical case of a 37-year-old female developing–immediately after chiropractic CSM therapy–bilateral VAD.
Orsini et al., 2019	Case report	Clinical case of 34-year-old women who underwent CSM by a chiropractor, followed by traumatic bilateral VAD, which resulted in vertebrobasilar stroke.
Tinel et al., 2008	Case report	Clinical case of 34-year-old male who underwent a cervical manipulation followed–by an alternate syndrome–with a right sensory motor deficit, cerebellar and pyramidal syndrome, and left deficit of cranial nerves.
Jeong et al., 2018	Case report	Clinical case of a patient which experienced a left posterior inferior cerebellar artery infarction and left VAD two weeks after a chiropractic CSM.
Horn, 1983	Case report	Clinical case of a 34-year-old male patient who underwent chiropractic CSM for the treatment of persistent headache, dizziness, and neck stiffness, and who, immediately after the procedure, developed a locked-in syndrome.
Ke et al., 2016	Case report	Clinical case of a 36-year-old male with a LIS consequent to bilateral VAD, developed after CSM.
Povlsen et al., 1987	Case report	Clinical case of a 36-year-old female who, one day after chiropractic CSM, developed an incomplete locked-in syndrome.
Hufnagel et al., 1999	Case series	Analysis of 10 cases of patients aged between 27 and 46 years which developed ischemic stroke secondary to vertebral or carotid artery dissection after chiropractic CSM.
Albuquerque et al., 2011	Retrospective observational study	Examination of 13 cases of patients presenting dissection of cervical and cranial segments of vertebral and carotid arteries after few hours to days of chiropractic manipulations.
Reuter et al., 2006	Retrospective observational study and survey	Analysis of the clinical pattern of 36 VAD cases associated to chiropractic neck manipulation reported over three years in Germany.
Haldeman et al., 2002	Retrospective review	Retrospective review of 64 medical legal cases of stroke temporally associated with CSM.
Thiel et al., 2007	Survey	The survey aimed to provide an estimate of the risk of serious and relatively minor adverse events following chiropractic CSM.
Cagnie et al., 2004	Prospective observational survey	Investigation of frequency of complications following spinal manipulations, even verifying their predictability.
Cagnie et al., 2005	Clinical trial	Cerebral blood flow changes investigation after CSM using 1-day split-dose Technetium 99 m–ethyl cysteinate dimer single photon emission computed tomography.

**Table 2:** Characteristics and summary and main findings of the analysed clinical studies.

Authors, Year	Type of Study	Summary	Main Findings
Reuter et al., 2006	Retrospective survey	Analysis of the clinical pattern of 36 VAD cases associated to chiropractic neck manipulation reported over three years in Germany.	Clinical symptoms consistent with VAD started in 55% of patients within 12 h after neck manipulation.
			VAD diagnosis was done in most cases using digital subtraction angiography (DSA), magnetic resonance angiography (MRA), or duplex sonography.
			90% of patients admitted to hospital had focal neurological deficits (among these, 11% had reduced level of consciousness).
			50% of patients were discharged after 20 ± 14 days with focal neurological deficits, one patient died, and one was in a persistent vegetative state.
			Risk factors associated with artery dissection were present in only 25% of patients.
Cagnie et al., 2004	Prospective survey	Investigation of frequency of complications following spinal manipulations, even verifying their predictability.	Reactions to spinal manipulation may be relatively common but are benign in nature and of short duration.
			Although it is difficult to label side effects as a risk, it is important to differentiate patients susceptible to side effects in order to inform them correctly.
Cagnie et al., 2005	Prospective study	Cerebral blood flow changes investigation after CSM using 1-day split-dose Technetium 99 m–ethyl cysteinate dimer single photon emission computed tomography.	Cerebellar hypoperfusion may occur after CSM. This could explain headache, dizziness, or nausea experienced by certain people after CSM.

## Data Availability

Not applicable.
